# Assessment of the quality of life of workers exposed to organic solvents: Study of 33 cases

**DOI:** 10.1192/j.eurpsy.2023.2047

**Published:** 2023-07-19

**Authors:** H. Ziedi, D. Brahim, H. Ben Said, A. Moussa, W. Ayed, S. Ernez, I. Youssef, N. Ladhari

**Affiliations:** Occupational pathology and fitness for work, Charles Nicolle Hospital, Tunis, Tunisia

## Abstract

**Introduction:**

Exposure to organic solvents (SO) is a significant occupational hazard in industrial settings. This can lead to neurobehavioural and physical effects that can affect the quality of life of workers

**Objectives:**

To assess, using a validated questionnaire, the quality of life of workers exposed to SO.

**Methods:**

Cross-sectional descriptive study conducted at the occupational medicine department of the Charles Nicolle Hospital in Tunis, which interested patients exposed to SO who had consulted for a medical opinion on fitness for duty during the period from January 1, 2017 to August 31, 2022. The data collection was carried out by a telephone call using the SF12 questionnaire. Socio-demographic and medical data were completed from medical records.

**Results:**

We identified 51 workers exposed to OS. Thirty-three employees agreed to answer the SF12 questionnaire, for a response rate of 65%. The average age was 44 8 years with a clear male predominance of 75%. The most represented sectors of activity were the automobile industry (34%), followed by the leather and footwear industry (15%) and the plastics industry (12%). The jobs most exposed to SO were manual workers (54%) and painters (9%). The median occupational seniority was 15[10; 23] years. Comorbidities were observed in 72% of employees. The average physical composite score was 48%. The average mental composite score was 49%. The average overall score was 49%. Average quality of life (overall SF12 score above 50) was noted in 60% of the population. Moderate disability (overall SF12 score between 30 and 39) was found in 18% of respondents. Twenty-one percent of workers had a severe disability (overall SF12 score below 30).

**Conclusions:**

In addition to socio-professional conditions, exposure to SO may be implicated in altering the quality of life of exposed workers. This implies the need to strengthen preventive measures in order to preserve the mental and physical health of these workers.

**Disclosure of Interest:**

None Declared

